# Climate Variability and Nonstationary Dynamics of *Mycoplasma pneumoniae* Pneumonia in Japan

**DOI:** 10.1371/journal.pone.0095447

**Published:** 2014-04-16

**Authors:** Daisuke Onozuka, Luis Fernando Chaves

**Affiliations:** 1 Department of Planning Information and Administration, Fukuoka Institute of Health and Environmental Sciences, Fukuoka, Japan; 2 Programa de Investigación en Enfermedades Tropicales, Escuela de Medicina Veterinaria, Universidad Nacional, Heredia, Costa Rica; 3 Institute of Tropical Medicine (NEKKEN), Nagasaki University, Nagasaki, Japan; University of Calgary & ProvLab Alberta, Canada

## Abstract

**Background:**

A stationary association between climate factors and epidemics of *Mycoplasma pneumoniae (M. pneumoniae)* pneumonia has been widely assumed. However, it is unclear whether elements of the local climate that are relevant to *M. pneumoniae* pneumonia transmission have stationary signatures of climate factors on their dynamics over different time scales.

**Methods:**

We performed a cross-wavelet coherency analysis to assess the patterns of association between monthly *M. pneumoniae* cases in Fukuoka, Japan, from 2000 to 2012 and indices for the Indian Ocean Dipole (IOD) and El Niño Southern Oscillation (ENSO).

**Results:**

Monthly *M. pneumoniae* cases were strongly associated with the dynamics of both the IOD and ENSO for the 1–2-year periodic mode in 2005–2007 and 2010–2011. This association was non-stationary and appeared to have a major influence on the synchrony of *M. pneumoniae* epidemics.

**Conclusions:**

Our results call for the consideration of non-stationary, possibly non-linear, patterns of association between *M. pneumoniae* cases and climatic factors in early warning systems.

## Introduction

*Mycoplasma pneumoniae (M. pneumoniae)* is a major cause of upper and lower respiratory tract infection and respiratory tract disease in humans, particularly in children [1]. This agent is estimated to be responsible for 15%–20% of all cases of community-acquired pneumonia (CAP), up to 40% of cases of CAP among children [2], and more than one-third of childhood cases of CAP requiring hospitalization [3].

As with *M. pneumoniae* infection, a recent study found quantitative evidence that the incidence of *M*. *pneumoniae* pneumonia increased significantly with increased average temperature and relative humidity [4]. However, it remains unclear whether local weather factors have non-stationary (not consistent over time) associations with *M*. *pneumoniae* infection. In this regard, interannual global climatic events related to the El Niño Southern Oscillation (ENSO) and Indian Ocean Dipole (IOD) have been associated with the transmission of a number of infectious diseases, including dengue [5], [6], malaria [7]–[9] and cholera [10]–[12]. However, the interannual cycles of *M*. *pneumoniae* infection have not been examined, and the possible clinical impact of these events on CAP remains unclear.

Wavelet analysis is used to identify non-stationarity associations between disease dynamics and exposure covariates, and is useful in the analysis of epidemiological time series [13]. In particular, wavelet analysis has been used to determine whether the presence of a particular periodic cycle at a given time in the incidence of a disease corresponds to the presence of the same periodic cycle at the same time in an exposure covariate [13]. Wavelet analysis is increasingly used in epidemiology to explore the spatial and temporal dynamics of diseases [14], [15].

*M. pneumoniae* pneumonia remains a major health risk in many parts of the world. A better understanding of its sensitivity to climate might aid in the development of a reliable climate-based prediction system for *M. pneumoniae* pneumonia epidemics, which might in turn lead to improvements in the current disease control program in Japan and other countries where *M. pneumoniae* pneumonia is endemic.

Here, we explored the time-varying relationships between climate variations and the monthly incidence of *M. pneumoniae* pneumonia between 2000 and 2012 in Fukuoka, Japan. To our knowledge, this is the first report to quantify the time-varying impact of climate factors on the number of *M. pneumoniae* pneumonia cases using cross-wavelet analysis.

## Materials and Methods

### Data Sources

The number of *M. pneumoniae* pneumonia patients in Fukuoka Prefecture, located in the southwest of Japan (Fig. S1), is reported on a weekly basis from 120 sentinel medical institutions [4]. Cases are defined by clinical factors in combination with laboratory tests. Clinical manifestations include fever, cough, malaise, and headache, sometimes with tracheobronchitis with fever and nonproductive cough [2], [16]. Several types of laboratory tests are used for the diagnosis such as culture, PCR, and serological methods. Serological methods include passive agglutination, complement fixation and ELISA, and a combination of PCR and serology is recommended for reliable diagnosis [17], [18]. We obtained clinical data that were recorded and reported by sentinel volunteers to the Fukuoka Institute of Health and Environmental Sciences, the municipal public health institute of the Fukuoka Prefectural Government. Monthly cases for *M. pneumoniae* pneumonia were calculated from the daily records based on the day of diagnosed. The population number and structure have changed little during the study period [19]. Additionally, the reporting system or case definition (diagnosis criteria) has not been changed.

The strength of the IOD was measured by the Dipole Mode Index (DMI), defined as the difference in sea surface temperature (SST) anomalies between the western (10°S–10°N, 50°–70°E) and eastern (10°S–0°, 90°–110°E) tropical Indian Ocean [20]. The DMI data were obtained from the Japan Agency for Marine-Earth Science and Technology (JAMSTEC) (http://www.jamstec.go.jp/frcgc/research/d1/iod/). The ENSO indices (i.e. Multivariate ENSO Index (MEI), Niño 1+2, Niño 3, Niño 4 and Niño 3.4) were also extracted from the National Oceanic and Atmospheric Administration (NOAA) Climate Prediction Center data (http://www.cpc.ncep.noaa.gov). The MEI is the six main observed variables over the tropical Pacific that predicts the occurrence of the ENSO; sea level pressure, zonal and meridional components of the surface wind, sea surface temperature, surface air temperature, and total cloudiness fraction of the sky [21]. Data of SSTs the tropical Pacific has been divided into a number of regions named Niño 1, 2, 3, 4, and 3.4; Niño 1 is the area defined by 80°–90°W and 5°–10°S, Niño 2 by 80°–90°W and 0°S–5°S, Niño 3 by 90°–150°W and 5°N–5°S, Niño 4 by 150°W–160°E and 5°N–5°S, Niño 3.4 by 120°–170°W and 5°N–5°S [21].

We also obtained data on daily average temperature, relative humidity and rainfall in the prefecture from the Japan Meteorological Agency. Monthly means for average temperatures, relative humidity and rainfall were calculated from the daily records.

The ethics committee of the Fukuoka Prefectural Government Health and Environmental Research Advancement Committee approved this study on December 27, 2006 (Reference Number: 18–3515). Patient records/information was anonymized and de-identified prior to analysis.

### Statistical Analysis

We examined the periodicity in the *M. pneumoniae* pneumonia incidence time series using cross-wavelet coherency analysis [13], [15]. This analysis provides the possibility of investigating and quantifying whether the presence of a particular frequency at a given time in *M. pneumoniae* pneumonia corresponds to the presence of the same frequency at the same time in a climate covariate. Cross-wavelet coherence significance was estimated using the method of Maraun and Kurths for a minimum time scale [22]. The Morlet Wavelet was used and the choice of minimum period of interest in the cycles was set at 6 months. A total smoothing window of 15 months was used to compute the cross-wavelet coherence. All statistical analyses were conducted using R statistical software, version 3.0.1.

## Results

A total of 23,325 (100%) cases of *M. pneumoniae* from 2000 to 2012 were included in our analyses, with 9,149 (39.2%) in children aged 0–4 years, 9,751 (41.8%) in those aged 5–9 years, 3,059 (13.1%) in those aged 10–14 years, and 1,366 (5.9%) in those aged 15 years or older.

**Figure 1:**
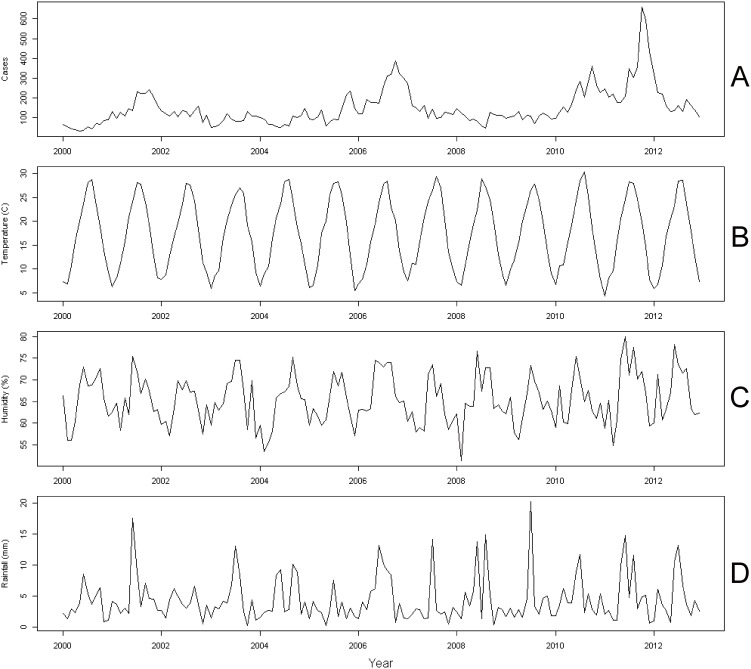
Monthly time series data in Fukuoka, Japan, 2000–2012. (A) *M. pneumoniae* pneumonia cases; (B) temperature; (C) relative humidity; and (D) rainfall.

The time series for the number of *M. pneumoniae* cases per month, ambient temperature, relative humidity and rainfall during the study period are shown in Figure 1. Analysis of reported cases of *M. pneumoniae* pneumonia by month revealed that the seasonal peak in cases was not identical from year to year (Fig. 1).

**Figure 2:**
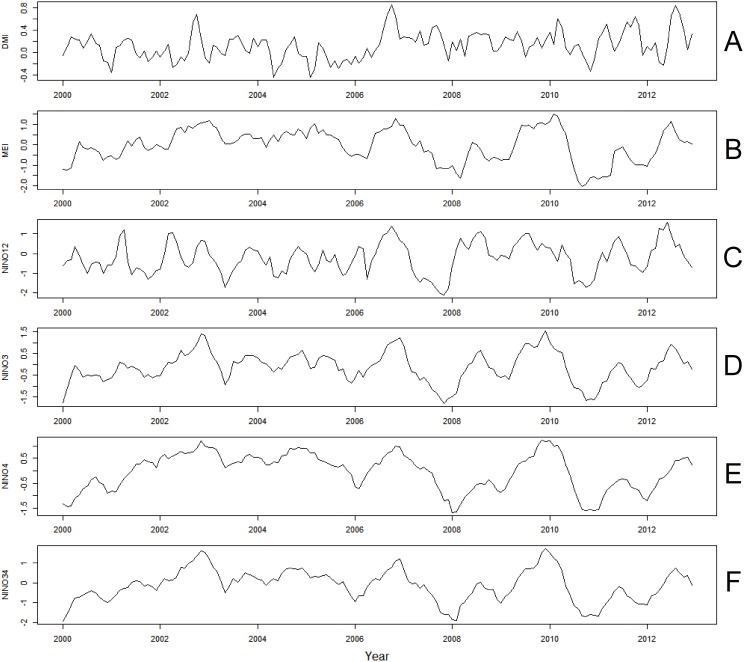
Monthly time series data for the global climatic indices (2000 to 2012). (A) DMI (dipole mode index); (B) MEI (multivariate ENSO index); (C) Niño 12 (ENSO index); (D) Niño 3 (ENSO index); (E) Niño 4 (ENSO index); (F) Niño 34 (ENSO index).

The time series for DMI and ENSO indices (MEI, Niño 1+2, Niño 3, Niño 4 and Niño 3.4) during the same period are shown in Figure 2. Strong positive IOD events (indicated by large DMI values) occurred in 2006 and 2012 and, in these years, the DMI peaked in October and August, respectively. Strong ENSO events (indicated by large MEI index values) were observed in 2006 and again in 2009–2010 (Fig. 2).

**Figure 3:**
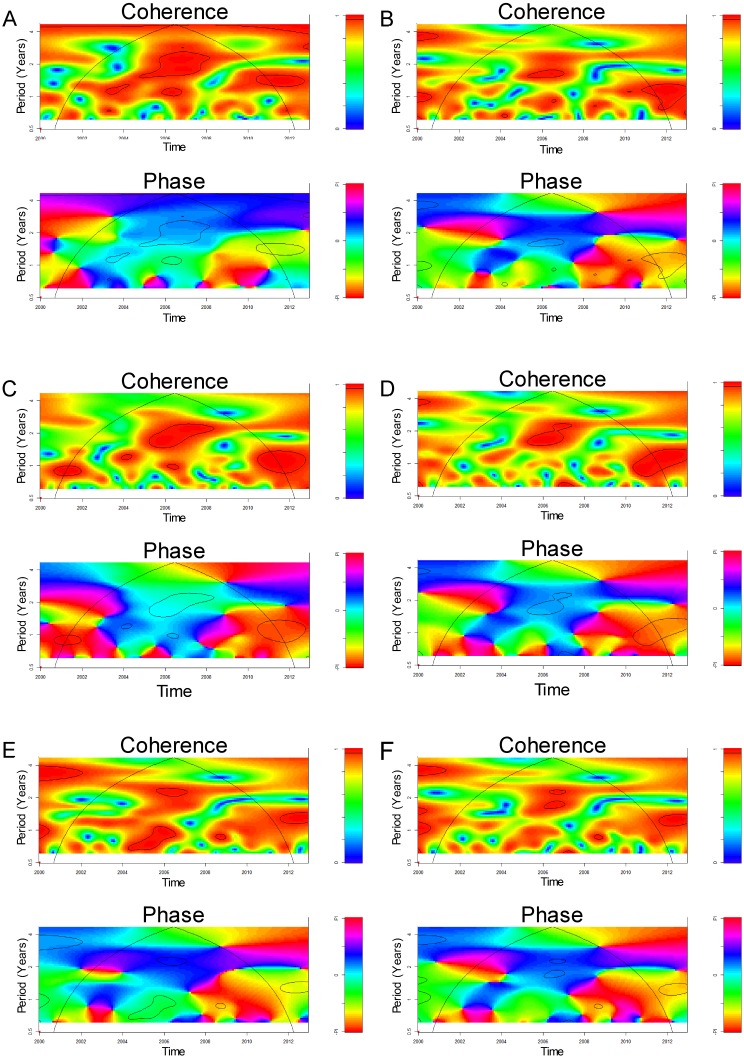
Cross-wavelet coherency and phase of the *M. pneumoniae* pneumonia time series with the global climatic indices. (A) DMI (dipole mode index); (B) MEI (multivariate ENSO index); (C) Niño 12 (ENSO index); (D) Niño 3 (ENSO index); (E) Niño 4 (ENSO index); (F) Niño 34 (ENSO index). Red regions in the upper part of the plots indicate frequencies and times for which the two series share variability. The cone of influence (within which results are not influenced by the edges of the data) and the significant coherent time-frequency regions (p<0.05) are indicated by solid lines. In cross-wavelet phase plots, colors correspond to different lags between the variability in the two series for a given time and frequency, measured in angles from -PI to PI. A value of PI corresponds to a lag of 16 months.

Cross-wavelet coherence and cross-wavelet phase analysis of the global climatic time series (DMI and ENSO indices) with *M. pneumoniae* cases are shown in Figure 3. Cross-wavelet coherency analysis revealed that *M. pneumoniae* cases were significantly (*p*< 0.05) coherent with DMI and ENSO indices at periods of around 1–2 years in 2005–2007 and 2010–2011, with DMI and ENSO indices leading the disease data, as shown by the cross-wavelet phases (Fig. 3).

**Figure 4:**
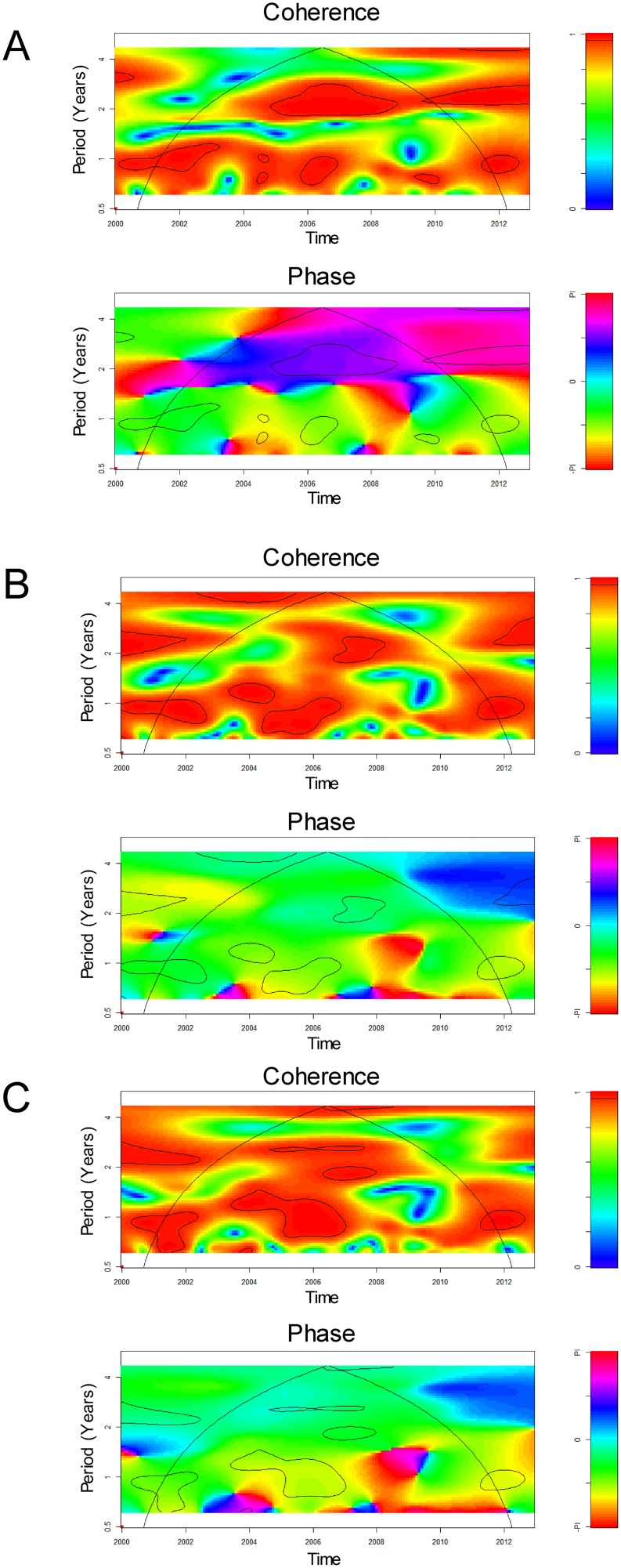
Cross-wavelet coherency and phase of the *M. pneumoniae* pneumonia time series with local weather indices. (A) Temperature; (B) relative humidity; and (C) rainfall. Red regions in the upper part of the plots indicate frequencies and times for which the two series share variability. The cone of influence (within which results are not influenced by the edges of the data) and the significant coherent time-frequency regions (p<0.05) are indicated by solid lines. In cross-wavelet phase plots, colors correspond to different lags between variability in the two series for a given time and frequency, measured in angles from -PI to PI. A value of PI corresponds to a lag of 16 months.

Cross-wavelet coherence and cross-wavelet phase analysis of the local climatic time series (temperature, relative humidity and rainfall) with *M. pneumoniae* cases are shown in Figure 4. Cross-wavelet coherency analysis revealed that *M. pneumoniae* cases were significantly (*p*<0.05) coherent with temperature, relative humidity and rainfall at periods of around 1–2 y, with temperature leading the disease data, as shown by the cross-wavelet phases (Fig. 4). All local climatic factors had non-stationary relationships with both DMI and ENSO indices (Fig. S2).

## Discussion

In this study, we found evidence that the monthly incidence of *M. pneumoniae* pneumonia cases was significantly associated with the dynamics of both the IOD and ENSO for the 1–2-year periodic mode in 2005–2007 and 2010–2011. This association was non-stationary and appeared to have a major influence on the synchrony of *M. pneumoniae* epidemics. These findings indicate that epidemic early warning systems should consider non-stationary, and possibly non-linear patterns of association between *M. pneumoniae* cases and climatic factors.

Anticipating the potential effects of changes in climate on the incidence and distribution of *M. pneumoniae* pneumonia requires a close understanding of the relationship between climate and disease dynamics [23]. Our present results show that the dynamics of *M. pneumoniae* pneumonia incidence are strongly associated with those of climate variables, including DMI and ENSO indices, with coherent cycles of around 1–2 years in 2005–2007 and 2010–2011. Consistent with this, an epidemiological study has suggested that El Niño events are associated with viral pneumonia hospitalizations [24]. In addition, a more recent study suggested that warming of the Indian Ocean relative to the Pacific might play an important role in modulating Pacific climate changes in the 20th and 21st centuries [25]. Another recent study has revealed a unique coupled mode over the Indo-Northwestern Pacific (NWP) warm pool during the boreal summer that arises from interaction between the Indian Ocean (IO) sea surface temperature and the Pacific-Japan (PJ) teleconnection pattern, and that this PJIO coupled mode can exist without ENSO but is efficiently excited by it [26]. Although childhood pneumonia, which is responsible for 17% of childhood deaths worldwide [27], is rarely mentioned in the context of climate change [28], our combined IOD and ENSO results demonstrate the importance of global climate factors in the prevalence of *M. pneumoniae* pneumonia infections.

Our study also found that the dynamics of *M. pneumoniae* pneumonia cases are strongly associated with local climate variables, temperature, relative humidity and rainfall, with coherent cycles of around 1–2 y in 2004–2007. Our results are consistent with a previous study which suggested that higher temperature and relative humidity might influence the increase in *M. pneumoniae* pneumonia cases after adjustment for potential confounding by seasonal and interannual patterns [4]. However, this previous study assumed that the association of temperature and relative humidity with *M. pneumoniae* pneumonia incidence was consistent over the study period. In contrast, our present findings suggest that the previous finding based on this assumption can be improved by assuming a non-stationary (not consistent over time) association, and by more accurately evaluating the possibility of a non-linear association between climatic covariates and *M. pneumoniae* pneumonia incidence.

A laboratory-based study has suggested that although the survival of airborne *M. pneumoniae* is determined by both temperature and relative humidity, the temperature response is mediated by relative humidity in that the effects of temperature are only observed if some water vapor is present [29]. Bacterial survival and virus stability in aerosols might be increased by higher humidity [28]. With respect to the effect of rainfall, an increase in time spent indoors because of increased rainfall will increase crowding and might lead to higher risk for contact transmission.

The finding for an association with a model which includes DMI and ENSO indices supports the recent proposal that large-scale climate indices may be more useful for forecasting than local climate variables [30]. Climate change is expected to have an enormous effect on the burden of infectious diseases due to the potential for increasing temperatures and changing rainfall patterns [31], and IOD and ENSO-related changes in temperature, rainfall, and other environmental factors might have both direct effects (e.g. extreme weather events) and indirect effects (e.g. changes in transmission and outbreaks of infectious diseases) on the incidence of *M. pneumoniae* pneumonia. Climate can affect the dynamics of infection in a host population through several linear and nonlinear pathways. Among these, it can affect several biological traits of parasitic organisms across their life cycle, from individual life histories to population dynamics [32]. Further, changes in climate and air quality substantially increase respiratory morbidity and mortality for patients with common chronic lung diseases such as asthma, chronic obstructive pulmonary disease (COPD) and respiratory tract infections [33]. While local climate is more likely to affect only the biological components of disease transmission, large-scale climate patterns might also influence contextual components of disease dynamics, such as population susceptibility.

Using cross-wavelet coherence and cross-wavelet phase analysis, we found that the effects of global and local climatic indices appeared at periods of around 1–2 y. These results may relate to the long incubation period of *M. pneumoniae*, and its relatively low transmission rate and late detection of outbreaks [34]. These characteristics hinder or prevent the efforts of public health officials and health-care providers in detecting *M. pneumoniae* pneumonia infections, and indicate the importance of developing more accurate and precise models of any effects of climate factors on disease risk, as well as further discussion of disease-specific issues.

A few limitations of this study warrant mention. First, not all cases in the community are represented in the surveillance data. This under-reporting can occur anywhere in the reporting chain, from the initial decision of a patient not to seek health care to the failure to record cases in the disease registry due to the mildness or lack of symptoms [35], and commonly occurs in closed and semi-closed communities [36]. However, we consider that this would have been unlikely to result in substantial bias because the degree of under-reporting is not likely to vary over time. Second, the participating sentinel medical institutions were recruited on a voluntary basis; however, this is unlikely to have posed a threat to the validity of the comparisons over time, which is the subject of this study.

Finally, with regard to the practical implications of our present findings, understanding the effects of climate factors on the epidemiology of infectious diseases is important for the planning of health services. These observed associations of climate factors with adverse health effects might represent a possible model or analog for the potential health impacts of future climate changes. Health services may need to prepare for the effects of climate change on the epidemiology of *M. pneumoniae* pneumonia through the implementation of preventive public health interventions. For instance, a recent study described the containment of an *M. pneumoniae* outbreak thanks to public health interventions that included communicating to the public the importance of respiratory hygiene, providing hand sanitizer in schools, and informing health-care providers about macrolide resistance [37]. Another study suggested that effective communications, coupled with university policies that help students adopt preventive health behaviors, including proper hand and respiratory hygiene, staying home and seeking medical care when ill, and refining health messages and communication methods to improve awareness of disease outbreaks among students, might reduce transmission of *M. pneumoniae* and the severe complications that can go with it [38]. These interventions reference the possibility of changes in behavior, such as people coming into closer contact with each other and thereby increasing the chance of person-to-person transmission.

In conclusion, this study found quantitative evidence that non-stationary associations have a major influence on synchrony between the monthly incidence of *M. pneumoniae* cases and the dynamics of both the IOD and ENSO for the 1–2-year periodic mode in 2005–2007 and 2010–2011. Our results call for the consideration of non-stationary, possibly non-linear patterns of association between *M. pneumoniae* cases and climatic factors in epidemic early warning systems.

## Supporting Information

Figure S1 Location of Fukuoka Prefecture on Kyushu Island, southwest of Tokyo, Japan.
(TIF)

Figure S2 Cross-wavelet coherency and phase of the temperature, humidity and rainfall time series with the DMI and with ENSO indices. (A) Temperature and DMI; (B) temperature and MEI; (C) temperature and Niño 1+2; (D) temperature and Niño 3; (E) temperature and Niño 4; (F) temperature and Niño 3.4; (G) relative humidity and DMI; (H) relative humidity and MEI; (I) relative humidity and Niño 1+2; (J) relative humidity and Niño 3; (K) relative humidity and Niño 4; (L) relative humidity and Niño 3.4; (M) rainfall and DMI; (N) rainfall and MEI; (O) rainfall and Niño 1+2; (P) rainfall and Niño 3; (Q) rainfall and Niño 4; and (R) rainfall and Niño 3.4. The coherency scale is from zero (blue) to one (red). Other technical details are presented in the legend of Fig. 3 and Fig. 4.
(TIF)
