# Supplementary figures and images for: Climate Variability and Nonstationary Dynamics of Mycoplasma pneumoniae Pneumonia in Japan
Source: PLoS One. 2014 Apr 16;9(4):e95447. doi: 10.1371/journal.pone.0095447 (PMC3989333; doi:10.1371/journal.pone.0095447)

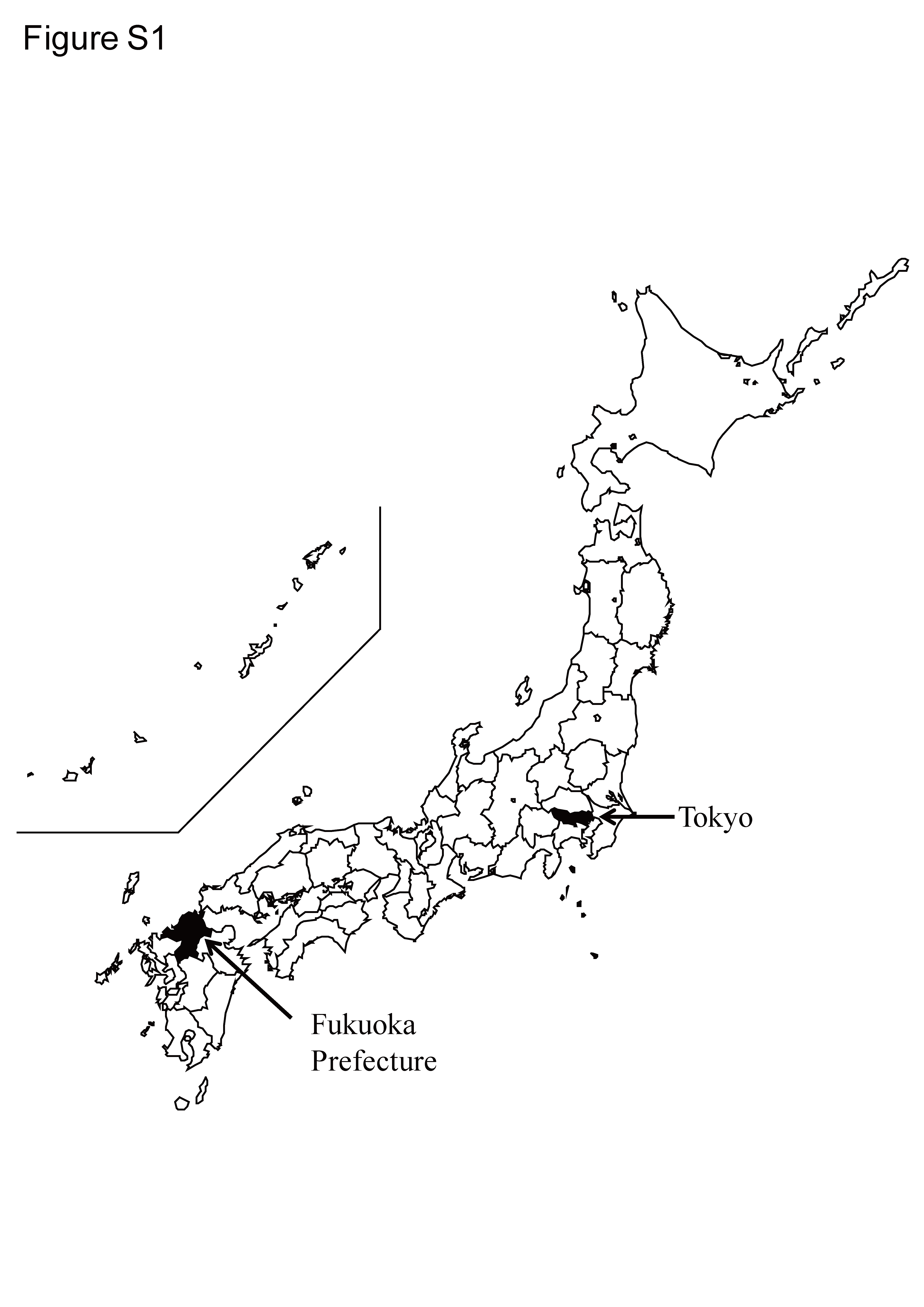

Supplement: Figure S1 — Location of Fukuoka Prefecture on Kyushu Island, southwest of Tokyo, Japan. (TIF) [file pone.0095447.s001.tif]

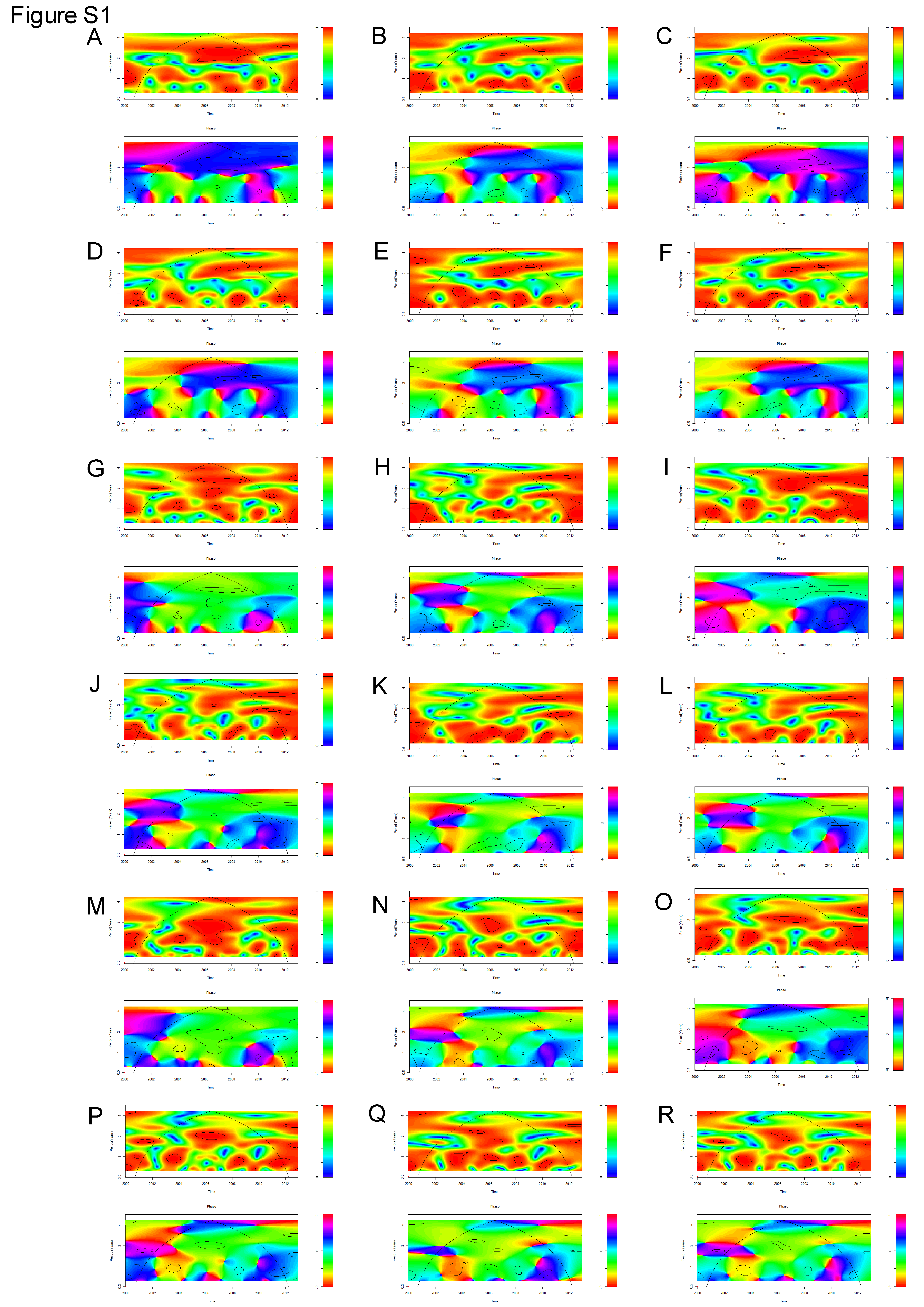

Supplement: Figure S2 — Cross-wavelet coherency and phase of the temperature, humidity and rainfall time series with the DMI and with ENSO indices. (A) Temperature and DMI; (B) temperature and MEI; (C) temperature and Niño 1+2; (D) temperature and Niño 3; (E) temperature and Niño 4; (F) temperature and Niño 3.4; (G) relative humidity and DMI; (H) relative humidity and MEI; (I) relative humidity and Niño 1+2; (J) relative humidity and Niño 3; (K) relative humidity and Niño 4; (L) relative humidity and Niño 3.4; (M) rainfall and DMI; (N) rainfall and MEI; (O) rainfall and Niño 1+2; (P) rainfall and Niño 3; (Q) rainfall and Niño 4; and (R) rainfall and Niño 3.4. The coherency scale is from zero (blue) to one (red). Other technical details are presented in the legend of Fig. 3 and Fig. 4. (TIF) [file pone.0095447.s002.tif]
